# A study on the impact and mechanism of action of public health education on the health of the migrant population: evidence from the 2018 China migrants dynamic survey

**DOI:** 10.3389/fpubh.2024.1308751

**Published:** 2024-02-09

**Authors:** Bo Dong

**Affiliations:** Wuhan University, Wuhan, China

**Keywords:** mobile population, health education, health impacts, mediating pathways, heterogeneity

## Abstract

**Background:**

China has the world’s largest mobile population. As mobility increases, ensuring the health protection of this population is receiving more attention. Strengthening public health education is a crucial measure to improve their health and achieve equal access to basic public health services in China. Previous research has demonstrated that public health education has an impact on the health of mobile populations. However, there has been limited investigation into the mediating pathways through which health education influences the health of mobile populations, and few studies have examined the heterogeneity of this effect.

**Objectives:**

The aim of this study was to analyze the impact of public health education on the health of the mobile population and its mechanism of action. Additionally, we aimed to explore the differences in this impact among different subdivided groups.

**Methods:**

This paper analyses the impact of public health education on the health of the mobile population using the 2018 China Migrant Dynamic Survey (CMDS) Data,. The data was cleaned and 4,034 people were included in the analysis. The study employed ordered logistic regression modeling to analyze the mediating pathways through which health education affects health. Additionally, binary logistic regression model, probit model, propensity score matching method and instrumental variables were used to verify the robustness of the results.

**Results:**

The self-assessed health status of the mobile population was good, and 82.10% of them accepted public health education. However, 17.89% of the mobile population did not receive any health education. Acceptance of health education can help improve the health status of the mobile population (OR = 1.178, 95% CI = 0.979–1.418). The study found that public health education can positively impact the health of mobile populations by influencing their health and hospitalization behaviors, as well as their social support. The analysis of heterogeneity revealed that the impact of public health education is more significant among rural, middle-aged, low-education, and low-income groups of the mobile population.

**Conclusion:**

Public health education can have a positive impact on the health of the migrant populations. To further improve health education for this group, it is necessary to actively promote the establishment of health records for the migrant population, to facilitate the contracting of family doctors by the migrant population, to improve the accessibility to hospitalization services, reduce the burden of hospitalization costs, and enhance social support. Simultaneously, it is essential to offer precise and varied health education to the migrant population based on their characteristics, to promote equity among diverse groups of individuals. These findings not only help to enrich theoretical research on health education for migrant populations and the health of migrant populations but also help to improve the level of public health education for migrant populations and improve the health protection of migrant populations.

## Introduction

In China, internal migrants are those whose current address does not match their household registration, also known as Hukou (1). The migrant population has made significant contributions to China’s rapid economic development (2, 3), and migration is often a crucial survival strategy for millions of people residing in rural areas (4). China has the world’s largest migrant population (5). According to the seventh national census data released by the Chinese government in 2021, the number of the migrants in China in 2020 was close to 380 million, which is an increase of 150 million from 2010, or an increase of nearly 70% in 10 years (6). As mobility increases, the health protection of the mobile population has become increasingly important. Research conducted by scholars from different countries indicates that the migrant population faces higher health risk due to their low level of education, poor working environment, and living conditions (7, 8), which makes them face higher health risks (9, 10). Moreover, public policies and social benefits in China are primarily based on hukou rather than the population residing in a particular area (11). As a result, the migrant population lacks the same level of social support and security as residents (12, 13), and the public health services of these migrant communities are inferior to those of permanent residents, which leads to higher health losses and worse health outcomes (12, 14, 15).

Public health education is a crucial aspect of disease prevention and health promotion. It is recognized worldwide as a strategic healthcare measure (16) and a fundamental component of public services (17). Effective public health education can improve self-health awareness and health literacy (18), promote healthy behaviors, and establish correct health concepts (19). To improve the health of the migrant populations, China has always prioritized public health education and considers it a crucial step toward achieving equal access to basic public health services and promoting health equity. The public health service project implemented in 2009, the project on equalization of basic public health and family planning services for the floating population implemented in 2013, and the action plan on health education and promotion for the floating population implemented in 2016, all of them have taken the provision of public health education for the floating population as an important aspect. Additionally, improving the health level of the floating population as an important goal.

In relation to public health education and the health of the migrant populations, relevant studies can be summarized in the following three aspects. Firstly, the current situation of the migrant population’s acceptance of health education and the factors that influence it. Regarding the current status of health education, the results of Yan et al. showed that the proportion of older migrant populations receiving public health education was low (20). Other studies have also indicated that health education needs to be more widely promoted and the coverage of health education should be expanded (21). Regarding the factors that influence access to public health education for mobile populations, age (22), income (23), medical insurance (24), and educational level (25) have been identified. Secondly, there is a relationship between public health education and the health of mobile populations, as it has been shown that public health education contributes to their well-being. Zhong et al. analyzed the impact of health education on the health of the mobile population using data from China’s 2017 China Migrant Dynamic Survey. They concluded that compared with those who did not receive public health education, receiving public health education resulted in a 4.1% improvement in self-health, and a reduction in the incidence of daily symptoms and diseases by 5.3 and 6.1% respectively, compared to those who did not receive public health education (17). Yan et al.’s findings also showed that receiving public health education increased the probability of self-assessed health improvement by 5.4–6.1% among the older adult mobile population (20). Thirdly, the path of action of public health education in influencing the health of the mobile population. Currently, only a few studies have analyzed the mediating mechanisms through which public health education affects the health of migrant populations. Li et al.’s study highlighted that public health education can affect the health of migrant populations through health records, family doctor contracting, and other pathways.

The studies above show that related scholars have extensively researched the relationship between health education and the migrant population’s health. However, there is still room for further expansion of related studies. Existing studies mainly focus on the health of the older adult, and there is a lack of expanding the research on all migrant populations. It is estimated that over 96% of the migrant population is under 60 years old. They usually have very different healthcare service needs and service utilization behaviors compared to older adults (26, 27). Therefore, the health education needs of this population may also differ from those of older adults, and attention should be paid to their specific needs. Additionally, previous studies have only examined the simpler mediating pathways and have not explored other pathways through which public health education affects the health of mobile populations. Finally, previous studies have not analyzed the heterogeneity of the impact of public health education on the health of different migrant populations.

Based on the above analysis, this study empirically analyzed the impact of public health education on the health of the mobile population using data from the nationally representative migrant dynamic survey data conducted by the National Health and Health Commission of China. The study also explored the mediating mechanism of health education’s impact on health by using structural equation modeling, and at the same time assessed the heterogeneity of the impact of public health education on the health of different subdivided groups in terms of various dimensions, such as age and income. At the same time, the heterogeneity of the impact of public health education on the health of different subgroups was assessed in terms of age, income, and other dimensions, to provide a reference for improving the public health education of the migrant population and enhancing their health. Compared to previous studies, this paper makes three significant contributions. Firstly, this paper not only analyzes the impact of public health education on the health of the mobile populations, but also further explores the mediating mechanism of the impact from multiple dimensions. Secondly, it examines the heterogeneity of the impact of public health education on the health of the mobile population from the four dimensions of urban and rural areas, age, level of education, and income, and provides a detailed examination of the differences in impact. Thirdly, this paper employs multiple methods to evaluate the impact of public health education on the health of different subgroups. Finally, this paper utilizes various methods to conduct robustness tests on the analysis results to enhance their accuracy and scientific validity.

## Materials and methods

### Data sources

China Migrants Dynamic Survey (CMDS) is a nationally representative survey conducted annually by the National Health and Health Commission of China since its establishment in 2009 (28). The data used in this paper is from the National Migrants Dynamic Survey carried out in 2018.

The CMDS is a reliable sample with a small sampling error (29). The survey adopts a hierarchical, multi-stage, and proportional-to-size PPS sampling method (30). In the first stage, the townships (towns and streets) were sampled according to the PPS method. In the second stage, village (neighborhood) committees were selected within the selected townships (towns and streets) according to the PPS method. In the third stage, individual respondents were selected from the chosen village (neighborhood) committees. This survey also employed rigorous methods to ensure data quality, including scientifically designed questionnaires, enumerators training, survey supervisors verification of questionnaires, and quality checks through telephone callbacks.

The survey respondents selected by CMDS were the floating population in 31 provinces and the Xinjiang Production and Construction Corps of China who were at least 15 years old, had resided in the inflow area for more than one month, and were not under the hukou of their districts (counties and cities). A total of 152,000 samples of the floating population were collected, resulting in a survey with a rich set of variables. The content of the CMDS survey covers basic demographic information, socio-economic conditions, utilization of medical services, public health care, and the social and economic status of the floating population. The CMDS survey also covers the basic demographic information, socio-economic status, medical service utilization, public health service utilization, and health of the mobile population. The final sample size for this study is 4,034 after the treatment of missing values and outliers.

### Variables

#### Dependent variables

This study employed self-assessed health as a measure of the mobile population’s health status. Previous research has shown that self-assessed health status aligns with an individual’s actual health level (31, 32) and can function as a versatile indicator applicable across various contexts, serving as a proxy for actual health status (33). The survey asked participants question posed was, “What is your health status?” with response options including “Not able to take care of yourself,” “Unhealthy but able to take care of yourself,” “Basically healthy,” and “Healthy.” For the purposes of this study, the responses were categorized into three groups: “1 = Unhealthy,” which includes the responses “Not able to take care of yourself” and “Unhealthy but able to take care of yourself,” “2 = Basically healthy,” and “3 = Healthy.” It’s important to note that self-rated health is treated as an ordinal variable, where higher values indicate better health.

#### Independent variables

The core independent variable in this study is the extent of public health education received. We constructed a binary variable (0–1) based on the survey question in the survey, “In the past year, have you received health education in the current residential community/unit?” The responses to this question included occupational disease prevention and control, infectious disease prevention and control, reproductive health and maternal and child health, chronic disease prevention and control, mental health, self-rescue during emergencies, and others. A value of 1 was assigned to respondents who reported receiving any of these types of education, while those who had not received any were assigned a value of 0.

#### Control variables

This study categorizes the control variables into three primary categories, namely individual characteristics, economic attributes, and healthcare insurance status, following Grossman’s Health Demand Model (34). Individual characteristics include gender (female = 0, male = 1), age (15–30 = 1, 31–45 = 2, 46–60 = 3,61 + =4), education level (illiterate = 1, primary school = 2, junior high school = 3, high school = 4, university and above = 5), marital status (unmarried = 0, married = 1), employment status (unemployed = 0, employed = 1), household registration (rural = 1, urban = 2), mobility range (within-city = 1, intercity within the province = 2, interprovincial = 3), and reasons for mobility (family = 1, work = 2, other = 3). Economic characteristics primarily consider household income, whereby income-related data is converted into rankings within each province (<20th percentile = 1, 20th-39th percentile = 2, 40th-59th percentile = 3, 60th-79th percentile = 4, and ≥ 80th percentile = 5) for data analysis. Healthcare insurance encompasses participation types, including Basic Medical Insurance System for Urban and Rural Residents (BMISURR) with a code of 1 and Basic Medical Insurance for Urban Employees(BMIUE) with a code of 2. Given the variations among provinces in economic and social environments, which may impact the health of mobile populations, this study also controls for the province of residence.

#### Mediating variables

Based on the existing research results (20, 29, 35, 36) and data availability, this study includes mediator variables such as health behavior and social support. Health behavior includes public health behavior and healthcare-seeking behavior. Public health behavior was measured by whether or not the participant had established a contract with a family doctor and whether or not to set up a health record was measured by the question “Have you established a resident health record in your local area?” The question of whether a health record has been established is “Have you established a health record in your local community?,” yes = 1, no = 0; the question of whether a family doctor has been contracted is “Have you contracted with a local family doctor?” Healthcare behaviors were measured by whether or not they were hospitalized, whether or not they were hospitalized locally, and whether or not they paid out-of-pocket hospitalization costs, with the question for whether or not they were hospitalized being “Have you been hospitalized yourself in the last year?,” Yes = 1, No = 0. The question for hospitalization is “In the last year, have you been hospitalized yourself?,” yes = 1, no = 0. The question for hospitalization in the local area is “Where were you hospitalized the last time?.” The corresponding question for personal out-of-pocket hospitalization costs is “How much did you pay out of the total cost of this hospitalization?.” To indirectly measure social support in the 2018 CMDS data, we used the willingness of the mobile population to stay. Social support can impact the willingness of the mobile population to stay (37), and receiving health education can also enhance their willingness to remain in the local area, making them more likely to stay in the inflow area (38). Therefore, it is reasonable to utilize the willingness to stay of the mobile population as a measure of social support. The survey question regarding the intention of the mobile population to stay in the local area is “Do you intend to stay in the local area in the coming period?” Yes = 1, No = 0. Combined with the above analysis, the definition and assignment of variables in this study are shown in Table 1.

**Table 1:** Definition of variables.

Variables			Definition
Dependent variables	Health		Unhealthy = 1; Basically Healthy = 2; Healthy = 3
Independent variables	Public health education		Yes = 1; No = 0
Control variables	Gender		Female = 0; Male = 1
	Age		15–30 = 1; 31–45 = 2; 46–60 = 3; 61 + =4
	Education		Illiterate = 1; Primary school = 2; Junior middle school = 3; Senior middle school = 4; University/college = 5
	Marriage status		Unmarried = 0; Married = 1
	Employment		Unemployed = 0; Employed = 1
	Household registration		Rural = 1; Urban = 2
	Range of migration		Intercounty = 1; Intercity = 2; Interprovince = 3
	Reasons for migration		Family = 1; Work = 2; Others = 3
	Household income ranking		Lowest(<percentile20) = 1; Lower(percentile20–39) = 2; Middle(percentile40–59) = 3; Higher(percentile60–79) = 4; Highest(≥percentile80) = 5
	Health insurance		BMISURR = 1; BMIUE = 2
Mediating variables	Utilization of public health services	Establishment of health records	No = 0; Yes = 1
		Family doctor signing	No = 0; Yes = 1
	Medical services utilization	Hospitalization	No = 0; Yes = 1
		Place of hospitalization	Household registration = 1; local (inflow) = 2
		Out-of-pocket hospitalization expenses	Continuous variable
	Social support	Residence intentions	No = 0; Yes = 1

### Statistical analysis

The data was analyzed using Stata22.0 software. Firstly, descriptive statistics were used to examine the data distribution of dependent, independent and control variables. Subsequently, we employed chi-square tests to investigate disparities in the health status of the mobile population across various characteristics. Secondly, significant independent variables identified in the univariate analysis were integrated into an ordered logistic regression model to evaluate the impact of public health education on the health of the mobile population. Thirdly, in exploring the mediating role of public health education on health outcomes, we utilized AMOS 25.0 to construct a structural equation model and perform standardized path testing. Bootstrapping was used to test the mediating effects. The test criteria of the structural equation model were the model fitting index, and the specific evaluation criteria were GFI, AGFI CFI > 0.9, and RMSEA<0.08, showing that the model had good validity. Lastly, the heterogeneity of public health education’s influence on the health of the migrant population was analyzed through four dimensions: urban–rural, age, education, and income. To ensure the robustness of our analysis, we extended it with binary logistic regression models, Probit models, propensity score matching methods and instrumental variable.”

## Results

### Characteristics of respondents

Table 2 demonstrates the results of descriptive statistics of the main variables in this study. Out of 4,034 respondents, 64.28% reported good health, while 24.84% reported basic health. The percentage of the mobile population receiving health education was 82.10%, but still, 17.90% of the mobile population did not receive health education. The education level of the migrant population is generally low, with only 38.47% having a university degree or higher, and 61.53% having a high school degree or lower. 53.54% of the migrant population have a rural household registration, and only 46.46% are from the cities. Analyzing the scope of mobility, the highest proportion is inter-provincial mobility, followed by intra-provincial inter-city and intra-city inter-county. 57.58% of the mobile population have an income below the median level. In terms of health insurance, 65.37% of the floating population are enrolled in the Basic Medical Insurance System for Urban and Rural Residents, while 34.63% are enrolled in Basic Medical Insurance for Urban Employees.

**Table 2:** Basic characteristics of respondents.

Variables		*N*	%
Health	Unhealthy	439	10.88
	Basically Healthy	1,002	24.84
	Healthy	2,539	64.28
Public health education	No	722	17.90
	Yes	3,312	82.10
Gender	Female	2,279	56.49
	Male	1755	43.51
Age	15–30	1,197	29.67
	31–45	1839	45.59
	46–60	759	18.82
	61+	239	5.92
Education	Illiterate	134	3.32
	Primary school	517	12.82
	Junior middle school	1,051	26.05
	Senior middle school	780	19.34
	University/college	1,552	38.47
Marriage	Unmarried	677	16.78
	Married	3,357	83.22
Employment	Unemployed	825	20.45
	Employed	3,209	79.55
Household registration	Rural	2,160	53.54
	Urban	1874	46.46
Range of migration	Intercounty	501	12.42
	Intercity	1,592	39.46
	Interprovince	1941	48.12
Reasons for migration	Family	665	16.48
	Work	3,303	81.88
	Others	66	1.64
Income	Lowest (<percentile20)	855	21.19
	Lower (percentile20–39)	730	18.10
	Middle (percentile40–59)	738	18.29
	Higher (percentile60–79)	805	19.96
	Highest (≥percentile80)	906	22.46
Health insurance	BMISURR	2,637	65.37
	BMIUE	1,397	34.63

### Differential analysis of health levels in mobile populations with various characteristics

Table 3 presents the results of our analysis, which focuses on the health of the mobile population as the dependent variable. The table reveals statistically significant differences in the health of mobile populations based on various factors, including gender, age, education, marital status, employment status, household registration, mobility range, reasons for mobility, income, and type of insurance coverage (all *p* < 0.05).

**Table 3:** Univariate analysis of health level of mobile population.

Variables		Health			*χ*^2^ value	*p*-value
		Unhealthy		Basically Healthy		Healthy			
		n	%	n	%	n	%	8.929	0.012
Gender	Female	249	56.72	526	52.50	1,504	58.00		
	Male	190	43.28	476	47.50	1,089	42.00		
Age	15–30	13	2.96	179	17.86	1,005	38.76	1159.153	0.000
	31–45	86	19.59	481	48.00	1,272	49.06		
	46–60	205	46.70	263	26.25	291	11.22		
	61+	135	30.75	79	7.88	25	0.96		
Education	Illiterate	79	18.00	35	3.49	20	0.77	916.047	0.000
	Primary school	173	39.41	166	16.57	178	6.86		
	Junior middle school	125	28.47	315	31.44	611	23.56		
	Senior middle school	46	10.48	187	18.66	547	21.10		
	University/college	16	3.64	299	29.84	1,237	47.71		
Marriage	Unmarried	53	12.07	145	14.47	479	18.47	16.110	0.000
	Married	386	87.93	857	85.53	2,114	81.53		
Employment	Unemployed	271	61.73	217	21.66	337	13.00	549.294	0.000
	Employed	168	38.27	785	78.34	2,256	87.00		
Household registration	Rural	325	74.03	561	55.99	1,274	49.13	96.776	0.000
	Urban	114	25.97	441	44.01	1,319	50.87		
Range of migration	Intercounty	57	12.98	150	14.97	294	11.34	22.401	0.000
	Intercity	202	46.01	354	35.33	1,036	39.95		
	Interprovince	180	41.00	498	49.70	1,263	48.71		
Reasons for migration	Family	137	31.21	172	17.17	356	13.73	147.836	0.000
	Work	278	63.33	807	80.54	2,218	85.54		
	Others	24	5.47	23	2.30	19	0.73		
Income	Lowest (<percentile20)	240	54.67	249	24.85	366	14.11	453.962	0.000
	Lower (percentile20–39)	90	20.50	202	20.16	438	16.89		
	Middle (percentile40–59)	56	12.76	180	17.96	502	19.36		
	Higher (percentile60–79)	37	8.43	183	18.26	585	22.56		
	Highest (≥percentile80)	16	3.64	188	18.76	702	27.07		
Health insurance	BMISURR	84	80.87	598	59.68	1955	75.40	544.006	0.000
	BMIUE	355	19.13	404	40.32	638	24.60		

### Examining the impact of public health education on the health of mobile populations

The independent variables used in the ordered logistic regression analysis were the statistically significant variables from the one-way analysis of variance. The dependent variable was the health of the mobile population. The results are shown in Table 4. The mobile population that received health education was more likely to have better health compared to those that did not receive public health education (OR = 1.178, 95% CI = 0.978–1.418), suggesting that access to public health education can promote better health among the mobile population. All control variables, the effects of all variables were significant, except for the effects of household registration, scope of mobility, and reason for mobility on the health of the mobile population, which were not significant. In terms of age, the health of the mobile population shows a decreasing trend as age increases. In terms of education level, increasing education level contributes to improving the health of the mobile population, with university and above having the most significant effect (OR = 4.097, 95% CI = 2.577–6.514). Higher income levels are also associated with better health outcomes for the mobile population, compared to those with the lowest income level. The mobile population with BMIUE have better health outcomes compared to those with BMISURR (OR = 1.266, 95% CI = 1.048–1.529).

**Table 4:** Ordered logistic regression results of public health education affecting the health of the mobile population.

Variables		β	SE	OR value	95% CI	*p*-value
Public health education	No (Reference)					
	Yes	0.164	0.094	1.178	0.979—1.418	0.083
Gender	Female (Reference)					
	Male	−0.115	0.079	0.856	0.734—0.998	0.048
Age	15–30 (Reference)					
	31–45	−0.836	0.109	0.434	0.350—0.537	<0.001
	46–60	−1.719	0.133	0.179	0.138—0.233	<0.001
	61-	−2.469	0.192	0.085	0.058—0.123	<0.001
Education	Illiterate (Reference)					
	Primary school	0.753	0.212	2.123	1.400—3.2192	<0.001
	Junior middle school	1.152	0.214	3.164	2.081—4.811	<0.001
	Senior middle school	1.296	0.228	3.655	2.339—5.712	<0.001
	University/college	1.410	0.237	4.097	2.577—6.514	<0.001
Marriage	Unmarried (Reference)					
	Married	0.408	0.118	1.504	1.193—1.895	0.001
Employment	Unemployed (Reference)					
	Employed	0.718	0.104	2.051	1.672—2.516	<0.001
Household registration	Rural (Reference)					
	Urban	−0.022	0.085	0.978	0.827—1.158	0.798
Range of migration	Intercounty (Reference)					
	Intercity	0.015	0.123	1.015	0.798—1.290	0.905
	Interprovince	0.052	0.129	1.054	0.817—1.359	0.687
Reasons for migration	Family = 1 (Reference)					
	Work = 2	−0.106	0.109	0.899	0.726—1.114	0.332
	Others = 3	−0.277	0.278	0.758	0.439—1.306	0.318
Income	Lowest (<percentile 20) (Reference)					
	Lower (percentile 20–39)	0.259	0.110	1.296	1.044—1.608	0.019
	Middle (percentile 40–59)	0.476	0.117	1.609	1.281—2.023	<0.001
	Higher (percentile60–79)	0.401	0.122	1.493	1.176—1.8954	0.001
	Highest (≥percentile 80)	0.531	0.129	1.701	1.318—1.194	<0.001
Health insurance	BMISURR (Reference)					
	BMIUE	0.236	0.096	1.266	1.048—1.529	0.014
Pseudo *R*^2^		0.208				
*N*		4,034				
Province of settlement		Control				

### Analysis of the mechanisms through which public health education impacts the health of the migrant population

This study examines the impact of public health education on the health of the migrant populations. The health of the mobile population is the dependent variable. Public health education is the independent variable, while public health service utilization and medical service utilization are the mediating variables. We employed AMOS 25.0 to establish the initial mediation model, conducted a single-step multiple mediation analysis, and evaluated the model’s fitness. The results, as shown in Table 5, indicates that the fitness *χ*^2^, with a value of 1.536 and a corresponding *p*-value of 0.078 (greater than 0.05), demonstrating a good fit with the sample data. Additionally, with an RMSEA value of 0.022 and values for CFI, GFI, and AGFI exceeding 0.90, various indices suggest that both the data and the overall model are well-suited for path estimation.

**Table 5:** Model fit index.

Evaluation indicators	Model results	Adaptation standards	Adaptation judgment
$\chi^{2}/df$	1.536	<3.00	Yes
$\chi^{2}$ -valued probability value *p*	0.078	>0.05	Yes
RMSEA	0.022	< 0.08	Yes
CFI	0.997	>0.90	Yes
GFI	0.997	>0.90	Yes
AGFI	0.973	>0.90	Yes
NFI	0.991	>0.90	Yes

**Figure 1:**
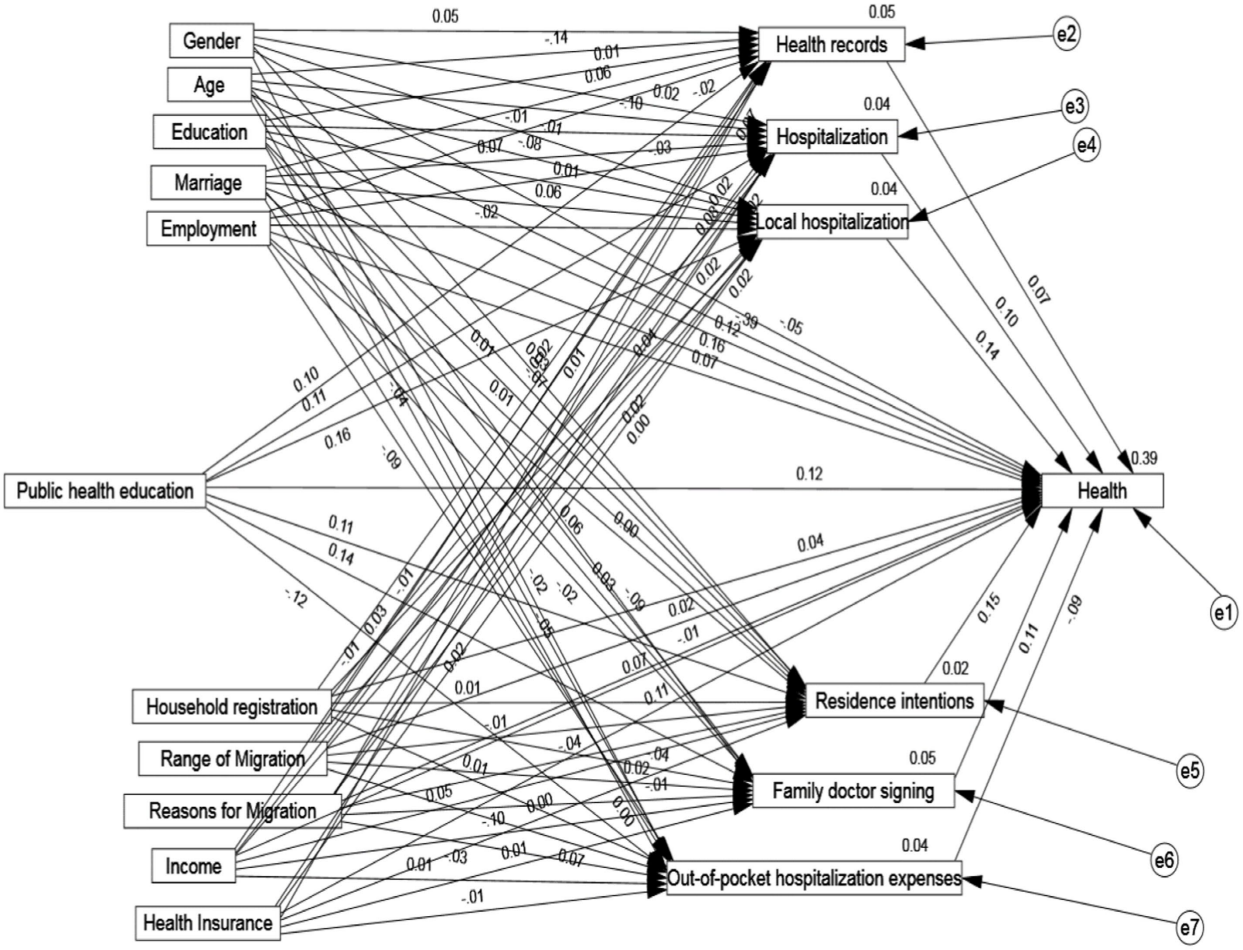
Mediating pathways through which public health education influences the health of mobile populations.

The next step involves estimating the coefficients of the mediated paths using the Bootstrap method. We set up 5,000 repeated random samples and 95% confidence intervals, and use the standardized regression coefficients as the criterion for judging to obtain the estimation of the unidirectional paths. As AMOS25 does not display significance levels for standardized results, we used unstandardized significance levels were used to indicate overall significance. Figure 1 demonstrates how public health education influence health, while Table 6 specifically presents the regression results with mediators such as health records, family doctor contracting, hospitalization behavior, hospitalization location, out-of-pocket hospitalization costs, and willingness to stay as mediators. The above results indicate that public health education has a significant positive direct effect on the health of the insured, with a coefficient of 0.124, and a significant positive effect on the establishment of a health record, with a coefficient of 0.105 at the 5% level of testing, while the health record has a significant positive effect on health, with a coefficient of 0.071 at the 1% level of testing, suggesting that there is an indirect effect from the health record on the health of the participants. These results suggest that family doctor contracting medicates the relationship between public health education and health. The study found that public health education has a significant positive effect on family doctor contracting with a coefficient of 0.139 at the 5% level, and family doctor contracting has a significant positive effect on health with a coefficient of 0.111, indicating that the mediating effect of family doctor contracting between public health education and health is valid. Public health education has a significant positive effect on the hospitalization behavior of the migrant population at the 1% test level with a coefficient of 0.11, and hospitalization behavior has a significant positive effect on health at the 5% test level with a coefficient of 0.098, indicating that insured hospitalization behavior mediates role in the effect of public health education on health.

**Table 6:** Results of the mediated path test of public health education affecting the health of the migrant population.

Path	Non-standardized coefficient	Standardization coefficient	SE	CR	*p*
Public health education → Health	0.112	0.124	0.022	5.039	<0.05
Public health education → Health records	0.104	0.105	0.029	3.563	<0.05
Health records → Health	0.065	0.071	0.022	2.959	<0.01
Public health education → Family doctor signing	0.139	0.139	0.029	4.71	<0.05
Family doctor signing → Health	0.1	0.111	0.022	4.588	<0.05
Public health education → Hospitalization	0.109	0.11	0.03	3.703	<0.05
Hospitalization → Health	0.089	0.098	0.022	4.085	<0.05
Public health education → Local hospitalization	0.156	0.156	0.029	5.283	<0.05
Local hospitalization → Health	0.125	0.137	0.022	5.722	<0.05
Public health education → Out-of-pocket hospitalization expenses	−0.121	−0.122	0.03	−4.106	<0.05
Out-of-pocket hospitalization expenses → Health	−0.08	−0.088	0.022	−3.672	<0.05
Public health education → Residence intentions	0.109	0.109	0.03	3.663	<0.05
Residence intentions → Health	0.137	0.151	0.022	6.353	<0.05

On the other hand, public health education has a significant positive effect on the location of hospitalization of the migrant population at the 5% significance, with a coefficient of 0.156. Additionally, the location of hospitalization has a significant positive effect on health at the 5% level of significance, with a coefficient of 0.137. This suggests that the choice of hospitalization location of the insured plays a mediating role in the effect of public health education on health. Meanwhile, public health education has a significant negative effect on out-of-pocket hospitalization costs of the migrant population with a coefficient of −0.122 at the 5% level of significance, while out-of-pocket hospitalization costs have a significant negative effect on health with a coefficient of −0.088 at the 5% level of test, indicating that out-of-pocket medical costs of the insured play an intermediary role in the effect of public health education on health. Finally, public health education has a significant positive effect on the willingness of the migrant population to stay in the country with a coefficient of 0.109 at the 5% level of the significance, while the willingness to stay in the country has a significant positive effect on health with a coefficient of 0.151 at the 5% level of test, indicating that the participant’s choice to stay in the relationship between public health education on health outcomes.

To examine how public health education impacts the health of migrant populations, this paper analyses the specific pathways. The results of this analysis are shown in Table 7. It can be seen that the indirect effect of the health record accounts for 5.88% of the total effect, the indirect effect of the family doctor contract accounts for 11.11% of the total effect, the indirect effect of the use of hospitalization services accounts for 8.20%, the indirect effect of hospitalization location is 14.39%. The study found that hospitalization cost had an indirect effect of 8.20% on the total effect, while willingness to stay had an indirect effect of 11.81%. The 95% CIs of the total, direct, and indirect effects of the six items did not include 0, indicating a significant mediating effect of the variables. The mediating role of health records, family doctor contracting, hospitalization service utilization, hospitalization location, out-of-pocket hospitalization costs and willingness to stay played a mediating role between public health education and health of the mobile population.

**Table 7:** Decomposition results of the mediating effect of public health education affecting the health of the mobile population.

Path	Total effect	*a*	*b*	Indirect effect	Direct effect	95% CI		Effect ratio
						Lower limit	Upper limit	
Public health education → Health records → Health	0.119**	0.104**	0.065**	0.007**	0.112**	0.002	0.015	5.88%
Public health education → Family doctor signing → Health	0.126**	0.139**	0.1**	0.014**	0.112**	0.007	0.024	11.11%
Public health education → Hospitalization → Health	0.122**	0.109**	0.089**	0.01**	0.112**	0.004	0.019	8.20%
Public health education → Local hospitalization → Health	0.132**	0.156**	0.125**	0.019**	0.112**	0.011	0.032	14.39%
Public health education → Out-of-pocket hospitalization expenses → Health	0.122**	−0.121**	−0.08**	0.01**	0.112**	0.003	0.02	8.20%
Public health education → Residence intentions → Health	0.127**	0.109**	0.137**	0.015**	0.112**	0.007	0.027	11.81%

***, **, * denote significant at the 1, 5, and 10% levels of significance.

### Heterogeneity analysis

To investigate the varying effects of public health education on the health status of different groups, this study categorized the mobile population by household registration, age, education level, and income, as shown in Table 8. Among the household registration groups, public health education was found to significantly enhance the health of both rural and urban mobile populations, with a greater impact observed among rural mobile populations. In the age groups, public health education has a significant impact on the health of middle-aged mobile populations. Within the education level subgroups, the effect of public education on the health status of mobile populations varies, with a significant impact observed among those with lower levels of education (high school education and below). In the income subgroups, the impact of public health education on the health of low-income mobile populations is significantly positive.

**Table 8:** Results of the heterogeneity analysis of the impact of public health education on the health of the mobile population.

Variables		Household registration		Age			Education		Income		
		Rural	Urban	Lower	Middle	Higher	High school and below	University/college	Lower	Middle	Higher
Public health education	β	0.289	0.267	−0.046	0.321	0.258	0.315	0.075	0.412	0.155	0.144
	SE	0.124	0.145	0.239	0.108	0.366	0.111	0.179	0.135	0.234	0.163
	OR value	1.336	1.306	0.956	1.379	1.294	1.369	1.078	1.154	1.168	1.150
	95% CI	1.048—1.702	0.983—1.734	0.598—1.531	1.116 — 1.704	0.632 — 2.649	1.103 — 1.702	0.759 — 0.532	1.159—1.969	0.738 — 1.848	0.839 — 1.588
	*p*-value	0.019	0.065	0.853	0.003	0.481	0.004	0.674	0.002	0.509	0.378
Control variables		Control	Control	Control	Control	Control	Control	Control	Control	Control	Control
Province of settlement		Control	Control	Control	Control	Control	Control	Control	Control	Control	Control
Pseudo *R*^2^		0.209	0.154	0.073	0.123	0.1332	0.185	0.076	0.204	0.154	0.111
N		2,160	1874	1,197	2,598	239	2,482	1,552	1,585	738	1711

### Robustness tests

#### Robustness test with replacement of dependent variables

To enhance the robustness of our findings, we initially tested them using the following three methods. Firstly, we assigned the dependent variable of self-assessed health was assigned as a dichotomous variable, i.e., “unable to take care of oneself” and “unhealthy but able to take care of oneself” were regarded as unhealthy and assigned the value of 0; “basically healthy” and “healthy” were regarded as healthy and assigned the value of 1, and then analyzed by binary logistic regression. “The results are shown in the first column of Model 1 in Table 9. Secondly, as the newly constructed dependent variable of self-assessed health is dichotomous, this paper further analyzes the impact of public health education on the health of the migrant population by using the Probit model. The impact of health education on the health of the migrant population and the results are shown in the second column of Model 2 in Table 9. Thirdly, the objective evaluation index of health, i.e., “whether it is sick or not (No = 0, Yes = 1)” is used as the measurement index of the health of the migrant population, and then analyzed by binary logistic regression, and the results are shown in the third column of Model 3 in Table 9. The study shows a positive correlation between public health education and the health of the migrant populations, with a significant level of at least 5%. This indicates the robustness of the results of this study.

**Table 9:** Robustness test results.

Variables		Health		
		Model 1	Model 2	Model 3
Public health education	β	0.335	0.178	0.138
	SE	0.155	0.084	0.057
	OR value	1.398	—	1.148
	*Z*-value	2.170	2.11	2.74
	95% CI	1.033—1.893	0.013—0.342	1.040—1.268
	*p*-value	0.030	0.034	0.006
Control variables		Control	Control	Control
Province of settlement		Control	Control	Control
Constant		1.8339 (1.277)	0.1479 (0.319)	0.2984 (0.049)
Pseudo *R*^2^		0.414	0.411	0.048
*N*		4,034	4,034	38,269

Standard error in parentheses.

#### Robustness test with different PSM methods

To mitigate potential endogeneity concerns between public health education and the health of the mobile population, we further validated the robustness of the results using propensity score matching (PSM) methods. We utilized both nearest-neighbor matching and radius matching techniques. The dependent variable was mobile population health, and we implemented 1:1 nearest-neighbor matching. The results are presented in Table 10. It is evident that, in comparison to the pre-matching results, the standardized deviations of most variables decreased (with most falling below 10%), indicating the acceptability of the matching outcomes.

**Table 10:** Variable error reduction analysis.

Variables	Sample	Average value		Standardized deviation (%)	Error reduction (%)	*t*-test	
		Test group	Control group			*t*-value	*p*-value
Gender	Before matching	0.430	0.443	−2.50%	52.5	−0.570	0.567
	After matching	0.434	0.440	−1.20%		−0.450	0.650
Age	Before matching	1.980	2.190	−24.30%	96.4	−5.800	0.000
	After matching	1.993	1.986	0.90%		0.350	0.725
Education	Before matching	3.796	3.582	17.50%	89.7	4.220	0.000
	After matching	3.786	3.808	−1.80%		−0.720	0.471
Marriage	Before matching	0.833	0.838	−1.60%	54.4	−0.360	0.717
	After matching	0.830	0.833	−0.70%		−0.270	0.784
Employment	Before matching	0.805	0.737	16.30%	96.1	3.930	0.000
	After matching	0.803	0.805	−0.60%		−0.260	0.796
Household registration	Before matching	1.473	1.428	9.20%	83.8	2.150	0.032
	After matching	1.468	1.475	−1.50%		−0.580	0.563
Range of migration	Before matching	2.338	2.435	−14.20%	84.9	−3.300	0.001
	After matching	2.348	2.363	−2.10%		−0.820	0.411
Reasons for migration	Before matching	1.855	1.822	8.20%	86.1	1.940	0.053
	After matching	1.854	1.859	−1.10%		−0.470	0.642
Income	Before matching	3.074	2.893	12.30%	96.9	2.900	0.004
	After matching	3.065	3.071	−0.40%		−0.150	0.881
Health insurance	Before matching	1.669	1.568	20.90%	88.7	4.960	0.000
	After matching	1.665	1.653	2.40%		0.930	0.350

Table 11 displays the outcomes of the average treatment effect (ATT) obtained using different methods. The results for ATT were 0.155 and 0.162 under both matching techniques, with corresponding *t*-values of 5.32 and 6.93, all significant at the 1% level. Furthermore, it was found that public education increased the likelihood of improving the health of the mobile population by 15.5 to 16.2%. This highlights the robustness of the conclusion that public health education has a significant and positive impact on the health of the mobile population.

**Table 11:** Propensity score matching estimation results.

Matching method	Test group	Control group	ATT	Standard error	*T*-value	*p*-value
Nearest neighbor match	2.560	2.405	0.155	0.029	5.32	0.000
Radius match (0.02)	2.560	2.389	0.162	0.029	6.93	0.000

#### Instrumental variables method

When mobile populations decide whether or not to receive public health education, their health status may also influence their decision. If they perceive their health status to be high, they may subjectively believe that the effect of health education is limited. This changes the relationship between health education and the health of the mobile population from a unidirectional influence of public health education on health to a bidirectional influence of both. Therefore, this study employs the instrumental variable method to address the endogeneity problem caused by bidirectional influence between public health education and the health level of the mobile population. Based on related research (39), this study chooses the variable of the number of community health education bulletin boards as an instrumental variable for public health education, as well as the number of health education bulletin boards can reflect both the degree of importance attached to public health education and the content and quality of publicity about public health education, and this variable is highly correlated with public health education, but does not have a direct impact on the health of the mobile population (39).

Table 12 presents the regression results obtained using inclusion of instrumental variables, where model (1) reports the results without control variables but with province-fixed effects, while model (2) is the result of regression with the inclusion of control variables and controlling for province fixed effects. From the results of the Wald test, the instrumental variable rejects the hypothesis of a weak instrumental variable and the original hypothesis of unrecognizability, and there is no endogeneity of this variable. From the regression results, after regression with instrumental variables, the coefficient of the health of the migrant population receiving community public health education is 5.5354, with a higher marginal effect is higher than that of the results of the benchmark regression model, which indicates that community health education can still significantly improve the health of the migrant population and that the benchmark regression underestimates the impact of community public health education on the health of the migrant population.

**Table 12:** Impact of public health education on the health of the mobile population.

Variables	Model 1	Model 2
Public health education	5.9069* (3.049)	5.5354** (2.517)
Control variables	Uncontrolled	Control
Province of settlement	Control	Control
*N*	242	242
Wald values	101.26	289.99
*R* ^2^	–	–

## Discussion

The aim of this study is to analyze the impact of public health education on the health of the migrant population and its mechanism of action, to provide support for better health improvement of the migrant population. This empirical evidence from China, as a developing country with the largest migrant population in the world, this empirical evidence from China will be of great practical significance. The study showed that 89.12% of the migrant population had good health, indicating the need for continuous improvement in their health. This is important as good health is essential for the effective utilization of their human capital (40). In terms of public health education, 82.10% of the mobile population received health education, while 17.90% did not have access to health education, which indicates that there is still a need to strengthen public health education for the mobile population.

The paper demonstrates that public health education can enhance the health of the migrant populations, even after controlling for province-fixed effects and adding control variables. This conclusion is consistent with previous studies. Golbeck et al.’s study showed that adult health education is an important way to improve health literacy (41), and the study by Santos et al. also concluded that health education improves health literacy and helps patients understand and comply with physician prescriptions (42). Health education is important for promoting health knowledge and advocating healthy lifestyles, particularly for migrant populations. It is important to ensure that public health education is appropriate and effective (20). Therefore, the migrant population should be fully encouraged to actively participate in public health education in their communities or units, and the coverage of health education should be continuously expanded to help the migrant population establish correct health concepts.

The study show that health records, family doctor contracting, hospitalization behavior, hospitalization location, hospitalization costs, and willingness to stay mediate the effect of public health education on the health of the migrant population. This suggests that public health education can improve the health of the migrant population by promoting better health behavior, medical care behavior, and social support. On the one hand, the above findings are consistent with existing studies, and also provide additional insights into how public health education can impact the health of the migrant population. For example, researchers used data from the China Migrant Population Dynamic Survey to analyze the impact of health education on the health of the migrant population and found that health records and family doctor contracting played a mediating role between health education and health (43). This result is consistent with the results of this study. However, this study analyzed the mechanism of action of health education on the health of the mobile population. It used a comprehensive set of variables and found that public health education could also improve health by influencing hospitalization behaviors, choice of hospitalization location, medical costs, and social support of the mobile population. As a crucial component of China’s basic public health service program, public health education can assist migrant populations in enhancing their health awareness and knowledge of disease transmission, among other things. This, in turn, can help achieve the objectives of disease prevention, health promotion, and improved quality of life by modifying their health behaviors (44). At the same time, public health education may influence the health of the mobile population by promoting their willingness to stay. This is because a robust public health service system is an important driving force for population migration (45, 46), and the public services in the city provide more opportunities and conveniences for the integration of the mobile population into the local area, which positively promotes their willingness to stay (47, 48). Therefore, the mobile population that has access to the public services is more likely to continue to stay in the inflow place (49, 50). In turn, social support represented by willingness to stay will have an impact on the health of mobile populations, because good social support can enhance the sense of belonging of mobile populations, satisfy their emotional needs in terms of informal support, and ultimately improve their physical and mental health (51). To improve the health of the mobile population firstly, it is recommended to actively promote the establishment of health records, continuously improve the management of health records, provide regular health services, and strengthen the management and dynamic monitoring of the health data. Secondly, the contracting and consulting work between family doctors and the migrant population should be comprehensively promoted to improve the family doctor contracting system for this group. This will allow family doctors to play their role as “gatekeepers” in health protection effectively. Improvements can be made to the use of hospitalization services for the floating population. This could be achieved by increasing the reimbursement ratio of medical insurance for hospitalization expenses and simplifying the reimbursement procedures. These changes would reduce the burden of hospitalization and improve the accessibility of hospitalization services for the floating population. Lastly, social support for the migrant population should be strengthened by encouraging the active participation of the migrant population in various public services to enhance their willingness to stay in the country, thereby providing conditions conducive to the improvement of their physical and mental health.

Further analysis of heterogeneity revealed that public health education was more effective in improving the health of the rural, middle-aged, low-educated, and low-income segments of the mobile population. The heterogeneity analysis results of these subdivided groups suggest that public health education for the mobile population should provide precise and diverse public services tailored to the characteristics of the subdivided target groups during policy design and resource allocation. This will enhance fairness among different groups of the population. It is worth noting that public health education has not had a significant impact on the health of migrant populations in both the younger and older age groups. This indicates that health education may still not have achieved its intended goal of improving health, and therefore health education should focus on the lower and the higher age groups of the migrant populations. To achieve the goal of accurate policy-making in health education, it is recommended to develop differentiated health education content based on the collection and analysis of health problems and health needs faced by these special populations. At the same time, innovative forms of health education, such as the Internet as an information dissemination medium play an important role in daily life, and many people will prioritize the use of the Internet to obtain health information and seek health support (52, 53). Therefore, the Internet can be utilized to deliver health education knowledge in a targeted manner to improve the acceptance and learning autonomy of the mobile population.

## Conclusion

This paper analyzes the impact of public health education on the health of the migrant population using data from the 2018 China Migrants Dynamic Survey (CMDS). It further examines the mediating effects of public health behaviors and healthcare-seeking behaviors on this impact, The study concludes as follows. Firstly, public health education plays an important role in improving the health of the migrant population, and more attention should be paid to the impact of health education on the migrant population. Secondly, public health education can affect health through their utilization of public health services, medical care behavior, and social support. Health records, family doctor contracts, hospitalization service utilization, and willingness to stay all play a mediating role in the impact of public health education on the health of the migrant population. Therefore, to further improve the health of the migrant population, it is necessary to improve the health management and family doctor contracting system, actively improve the accessibility of hospitalization for the migrant population, and reduce the personal burden of hospitalization, as well as strengthen the social support for the migrant population. Finally, there is heterogeneity in the impact of public health education on the health of the migrant populations. To improve its impact, health education content should be enriched and innovative forms of health education should be developed to meet the specific health needs of different groups.

### Limitations

It should be noted that this study still has certain limitations. Firstly, self-assessed health indicators are used to reflect the health status of individuals, which may introduce subjectivity. Further studies should aim to enrich and improve the measurement indicators. Secondly, there may be other mediating paths for public health education to influence the health of the mobile population, this study analyzes the mediating role of three paths, namely, public health service utilization, hospitalization service utilization, and social support, based on the availability of data from the 2018 CMDS. In the future, other paths can be explored with the abundance of data, such as lifestyle factors, environmental influences, social support, and psychological related variables. To more comprehensively investigate the impact of health education on the migrant population’s health, the analysis should include support and psychologically related variables. Thirdly, this study may have omitted other factors affecting health due to its complexity. Therefore, when selecting the factors affecting the health of the migrant population through public health education, it is important to consider other potential influences for inclusion in future analyses. Fourthly, because we used data from the 2018 Migrant Population Dynamic Surveillance Survey, the cross-sectional data made the amount of data in this study relatively small, which may limit the generalizability and statistical power of the findings, resulting in findings that are not representative of a larger population. In addition, the limited data may also affect the ability to detect significant effects or differences, leading to uncertain interpretations. Future studies, as data become more abundant, may use longitudinal data to expand the dataset, employ supplemental statistical methods, or consider alternative study designs that are more appropriate to the limited data situation to enhance the findings and improve the representativeness and scientific validity of the results.

## Data availability statement

Publicly available datasets were analyzed in this study. This data can be found here: This study was based on a publicly available database. Dataset available via the Migrant Population Service Center, National Health Commission, China. The datasets generated and/or analyzed during the current study can be found at: https://www.chinaldrk.org.cn/.

## Ethics statement

Ethical approval was not required, as this study was a secondary analysis conducted using public data sets from the CMDS that did not include identifiable personal information. Each volunteer participant obtained a written informed consent based on inclusion criteria. For illiterate participants, informed consent was obtained from their next of kin/legally authorized representative. The authors declare that all methods were carried out in accordance with relevant guidelines and regulations.

## Author contributions

DB collected, cleaned and prepared the data, analyzed and interpreted the data, drafted the manuscript and made subsequent revisions, read and approved the final manuscript.
